# Probiotic *lactobacillus* and estrogen effects on vaginal epithelial gene expression responses to *Candida albicans*

**DOI:** 10.1186/1423-0127-19-58

**Published:** 2012-06-20

**Authors:** R Doug Wagner, Shemedia J Johnson

**Affiliations:** 1 Microbiology Division, National Center for Toxicological Research, 3900 NCTR Rd, Jefferson, AR, 72079, USA

**Keywords:** Probiotic, Epithelial cells, Gene expression, Signal transduction genes, Candidiasis, Estrogen

## Abstract

**Background:**

Vaginal epithelial cells have receptors, signal transduction mechanisms, and cytokine secretion capabilities to recruit host defenses against *Candida albicans* infections. This research evaluates how probiotic lactobacilli affect the defensive epithelial response.

**Methods:**

This study used quantitative reverse transcription-polymerase chain reaction assay (qRT-PCR), flow cytometry, and a multiplex immunoassay to observe changes in the regulation of gene expression related to cytokine responses in the VK2 (E6/E7) vaginal epithelial cell line treated with 17β-estradiol, exposed to probiotic *Lactobacillus rhamnosus* GR-1® and *Lactobacillus reuteri* RC-14® and challenged with *C. albicans*. Data were statistically evaluated by repeated measures analysis of variance and paired t-tests where appropriate.

**Results:**

*C. albicans* induced mRNA expression of genes related to inflammatory cytokine responses associated with nuclear factor-kappa B (NF-κB) and mitogen-activated protein kinase (MAPK) signal transduction pathways. 17β-estradiol suppressed expression of interleukin-1α (IL-1α), IL-6, IL-8, and tumor necrosis factor alpha (TNFα) mRNA. Probiotic lactobacilli suppressed *C. albicans*-induced nuclear factor-kappa B inhibitor kinase kinase alpha (Iκκα), Toll-like receptor-2 (TLR2), TLR6, IL-8, and TNFα, also suggesting inhibition of NF-κB signaling. The lactobacilli induced expression of IL-1α, and IL-1β mRNA, which was not inhibited by curcumin, suggesting that they induce an alternate inflammatory signal transduction pathway to NF-κB, such as the mitogen activated protein kinase and activator protein-1 (MAPK/AP-1) signal transduction pathway. Curcumin inhibited IL-13 secretion, suggesting that expression of this cytokine is mainly regulated by NF-κB signaling in VK2 cells.

**Conclusions:**

The results suggest that *C. albicans* infection induces pro-inflammatory responses in vaginal epithelial cells, and estrogen and lactobacilli suppress expression of NF-κB-related inflammatory genes. Probiotic lactobacilli may induce IL-1α and IL-1β expression by an alternate signal transduction pathway, such as MAPK/AP-1. Activation of alternate signaling mechanisms by lactobacilli to modify epithelial cell cytokine production may be a mechanism for probiotic modulation of morbidity in vulvovaginal candidiasis.

## Background

The vaginal microbiota is one of the first lines of defense against vulvovaginal candidiasis (VVC). The normal vaginal microbiota is predominantly populated by *Lactobacillus crispatus, Lactobacillus jensenii*, and *Lactobacillus iners*[1], which tend to suppress growth of other bacterial species by production of lactic acid and antimicrobial products [2]. Some *in vivo* experiments have shown that the strains *L. rhamnosus* GR-1 and *L. reuteri* RC-14 may be effective treatments for VVC [3]. These organisms may partially affect resistance to yeast infections by modulating the pro-inflammatory responses of vaginal epithelial cells to the fungus.

The vaginal epithelial cell is the next line of defense against *Candida* spp. in the vagina. These epithelial cells have anti-*Candida* spp. activity, which is reduced in women with recurrent VVC [4]. The predominant mode of protection against VVC has shifted in the estimation of some scientists from adaptive immunity to innate immunity, which may be responsible for symptomatic presentation of the disease [5]. The vaginal epithelial cell can be considered part of this innate defense system, as it is capable of producing pro-inflammatory cytokines. These cells are known to send Langerhans cell recruitment signals in the form of the CCL20 chemokine [6] and they also express other immune recruitment molecules [7]. Epithelial cells of the human genitourinary tract respond to microbial surface molecules with Toll-like receptors-2, 4, and 6 that initiate transcription of pro-inflammatory cytokines, such as interleukin-8 (IL-8), IL-6, IL-1α, IL-1β, and tumor necrosis factor alpha (TNFα) by way of the nuclear factor-kappa B (NF-κB) signal transduction pathway [8,9]. Other microbial surface molecule pattern recognition molecules and signal transduction pathways may also be involved in pro-inflammatory epithelial cell responses. Recognition of surface molecular patterns of probiotic bacteria by VEC may account for some of their ability to modify host responses to *C. albicans*.

Estrogens appear to affect the recruitment of host defenses to *C. albicans* by epithelial cells of the female reproductive tract. For example, primary uterine epithelial cells respond to estrogen with decreased secretion of lipopolysaccharide-induced cytokines, IL-6, IL-8, and macrophage migration inhibition factor [10]. Expression of the regulatory protein NF-κB is also decreased in these cells by estrogen, which may cause decreased expression of cytokine genes, such as IL-6, IL-8, IL-1α, IL-1β, TNFα, and others under its regulation. Thus, estrogen may be an important factor affecting the barrier and host defense recruitment functions of the vaginal epithelium as well, and its role must be assessed in conjunction with studies of probiotic lactobacilli effects.

The present study was done to evaluate effects of probiotic lactobacilli on VVC at the point of fungal contact with vaginal epithelial cells with estrogen present in the form of 17β-estradiol. The effects of probiotic lactobacilli on how the VK2 cell model of vaginal epithelial cells respond to contact with *C. albicans*, with and without the influence of estrogen, was studied at the level of expression of genes for intracellular signal transduction mechanisms and intercellular signaling by cytokines.

## Methods

### Microbial strains and growth conditions

*Candida albicans* strain B 311 (ATCC 32354) was grown aerobically overnight in Sabouraud’s dextrose broth (Thermo Fisher Scientific, Houston, TX) at 37 °C. *L. rhamnosus* GR-1® and *L. reuteri* RC-14® (provided by Dr. Gregor Reid, Urex Bioscience, London, Ontario) were grown anaerobically in MRS broth (Thermo Fisher) at 35 °C.

### Culture and microbial challenge of VK2 cell line

The VK2 (E6/E7) vaginal epithelial cell line (VK2) [11], purchased from the American Type Culture Collection, Rockville, MD, as product CRL-2616, was grown to confluence as polarized monolayers on 35 mm polycarbonate inserts [7,12] in keratinocyte-serum free medium (K-SFM) containing 5 ng/ml recombinant epidermal growth factor and 50 μg/ml bovine pituitary extract (Invitrogen Corporation, Grand Island, NY) and some experimental groups contained 10 nM 17 β-estradiol added to the basolateral medium [13]. Genital tract secretions (GTS) medium [14] was used on the apical side of the VK2 cell monolayers. The cells were grown at 37 °C with a 5% CO_2_ atmosphere and 100% humidity. Confluence, polarization, and differentiation of the VK2 cell monolayers were assessed by measurement of trans-epithelial electrical resistance (TEER) with an EVOM device (World Precision Instruments, Inc., Sarasota, FL). Viability of the VK2 cell monolayers were assessed by measurement of lactate dehydrogenase (LDH) released into the basolateral medium after experimental treatments using a commercial assay kit (Takara LDH cytotoxicity detection kit, Thermo Fisher). Probiotic lactobacilli were added at 1 X 10^7^ CFU/ml to the apical culture chambers 4 hr before an 18 hour challenge with 2 X 10^6^ CFU/ml *C. albicans*. The six experimental groups compared in this study were: (1) VK2 cells grown 18 hr with K-SFM medium on the basolateral sides and GTS medium on the apical sides of the culture inserts, (2) VK2 cells cultured in the same media and challenged with 2 X 10^6^ CFU *C. albicans*, (3) VK2 cells with 10 nM 17β-estradiol in the basolateral medium, (4) VK2 cells in the same media with 10 nM 17β-estradiol added to the basolateral medium and challenged with 2 X 10^6^ CFU *C. albicans*, (5) VK2 cells incubated 22 hours with 1 X 10^7^ CFU of both *Lactobacillus* strains in the apical GTS medium, and (6) VK2 cells cultured with 10 nM 17β-estradiol added to the basolateral medium and pre-incubated 4 hours with 1 X 10^7^ CFU of both *Lactobacillus* strains before challenge with 2 X 10^6^ CFU *C. albicans.* The final incubations were 18 hours after *C. albicans* challenge.

### Real-time RT-PCR gene expression profiling

Total cellular RNA samples from VK2 cell monolayers were isolated using RNeasy Protect™ total RNA isolation kits (Qiagen, Inc., Valencia, CA). The RNA samples were reverse-transcribed with the RT^2^ PCR Array™ first strand kit (SABioscience, Fredrick, MD) and the resulting cDNA were applied to real-time PCR using RT^2^-Profiler™ kits (SABioscience) for detection in a BioRad (Hercules, CA) Pci,Q5 instrument. The RT^2^-Profiler™ kits used in the study were: PAHS-011A Human Inflammatory Cytokines and Receptors, PAHS-052A Human Innate and Adaptive Immune Responses, PAHS-025A Human NF-κB Signaling Pathway, PAHS-077A Human Inflammatory Response and Autoimmunity, PAHS-014A Human Signal Transduction Pathway Finder; together, they measured mRNA levels of 145 genes associated with innate immune cell signaling. The PAHS-999A Human RT^2^ RNA QC PCR Array was used to validate the quality of the cDNA products prior to real-time PCR analysis. The RT^2^ PCR Array™ products contain primers for housekeeping genes: beta-2 microglobulin, hypoxanthine phosphoribosyl transferase 1, ribosomal protein L13a, glyceraldehyde-3-phosphate dehydrogenase, and beta actin*,* which are used by the analysis software as internal controls. Results of the PCR array experiments were analyzed with online software at SABioscience to determine the key signal transduction pathways and immune system interaction genes affected by *C. albicans*, 17β-estradiol, and the probiotic *Lactobacillus* spp. strains. The software calculated fold changes in mRNA concentrations using the formula: 2^-ΔCt^ test sample/2^-ΔCt^ control sample. The average ΔCt values of test (n = 6) samples were corrected for average ΔCt values of 5 housekeeping genes before inclusion in the formula. Comparisons of average ΔCt values of test (n = 6) samples were statistically significant according to the t-test at P < 0.05. Average changes of two-fold or more in expression of genes were considered significant.

### Flow cytometric analysis of gene products

Flow cytometry with an Accuri C6 cytometer (Accuri Cytometers, Ann Arbor, MI) using fluorescently labeled antibodies from Santa Cruz Biotechnology, Santa Cruz, CA and Biolegend, San Diego, CA, was used to compare the phenotypic responses in the VK2 cells to the genotypic responses measured with the quantitative real-time RT-PCR profiles.

VK2 cells incubated with 10 nM 17β-estradiol and an equal mixture of probiotic strains or with 10 nM 17β-estradiol alone were challenged with *C. albicans*, as described above and then stained with fluorescent antibodies for surface receptors prior to analysis by flow cytometry. Ten thousand events were accumulated from each analysis for comparison between experimental groups. Many of the signal transduction antigens of interest are only expressed intracellularly, so Santa Cruz Biotechnology intracellular flow cytometry staining kits were used to prepare the cells for analysis.

Extracellular and intracellular staining methods were used as described by the antibody manufacturers. Antibodies were applied at concentrations recommended by the manufacturers, usually 1 μg/ml for 1 X 10^6^ cells. The stained cells were suspended in 1% paraformaldehyde PBS solution (Boston Bioproducts, Inc., Worcester, MA).

The flow cytometer was calibrated with SPHERO™ Rainbow 8-Peak and Allophycocyanin 6-Peak calibration particles (Spherotech, Inc., Lake Forest, IL). After 10,000 cells were counted in a sample, a density plot of measurements from side scatter *versus* the appropriate fluorescence channel was analyzed with a polyhedral gate to determine the percentage of stained cells. Averaged relative fluorescence values were compared to assess significant changes in expression of specific genes at the level of protein expression.

### Quantitative cytokine immunoassay

A MultiBead^®^ multiplex immunoassay (Enzo Life Sciences, Ann Arbor, MI) was used to quantify cytokines excreted into the basolateral growth media by VK2 cells. Using the manufacturer’s instructions, culture supernatants were incubated with allophycocyanin -labeled 4 μm and 5.4 μm anti-human cytokine antibody-coated capture beads that were washed in 96 well filter plates on a vacuum manifold (Enzo) set at 100 mbar vacuum. The beads were incubated with phycoerythrin-labeled streptavidin and washed on the vacuum manifold. The beads were analyzed in a C6^®^ digital flow cytometer (Accuri Cytometers) that was calibrated with Rainbow 8-peak® and Allophycocyanin 6-Peak Calibration Particles® (Spherotech). Each size bead population was gated with forward and side scatter dot plots and individual allophycocyanin fluorescence intensities corresponding to specific anti-cytokine antibody were gated with an emission detector filtered at 675 nm (FL-4) after excitation with a 640 nm laser. Fluorescence of phycoerythrin was used to obtain quantitative information by excitation with a laser at 488 nm and detection at 585 nm (FL-2). Results were obtained from a standard curve in Assay Designs MultiBead^®^ analysis software using standards supplied by the kit manufacturer (Enzo).

### NF-κB signal transduction pathway inhibition

The inhibitor of NF-κB nuclear transduction, curcumin (Imgenex, San Diego, CA), was used to assess whether probiotic effects on VK2 cell cytokine responses were mediated exclusively by the NF-κB signal transduction pathway. VK2 cells grown to confluence on polyester inserts, as described above, were divided into four experimental groups: (1) VK2 cells, (2) VK2 cells + 100 μM curcumin, (3) VK2 cells + lactobacilli and, (4) VK2 cells + lactobacilli + 100 μM curcumin. The apical medium was GTS [14]. For groups 2 and 4, the basolateral media were replaced with curcumin solution and incubated 4 hr at 37 °C. Groups 3 and 4 received 1 X 10^7^ CFU/ml of the *Lactobacillus* mixture and were incubated 18 hours. After incubation, media from the inserts (apical) and the wells (basolateral) were centrifuged at 10,000 x *g* for 2 min and the supernatants were assayed with the MultiBead^®^ multiplex immunoassay to determine cytokine concentrations.

### Analysis of data

The mean fold differences in expression of genes detected by the RT^2^PCR arrays and curcumin inhibition of cytokine production assays from 6 independent experiments per treatment group were compared for statistical significance with the Repeated Measures One-Way Analysis of Variance and Newman-Keuls post tests [15] using Prism v. 5.0 software (GraphPad Software, San Diego, CA). Paired comparisons of flow cytometry and cytokine immunoassay data were made with paired t-tests in Prism v. 5.0 software to assess statistically significant differences. Statistical significance was defined as P < 0.05.

## Results

### Epithelial permeability, but not viability was affected by estrogen and microbes

**Figure 1:**
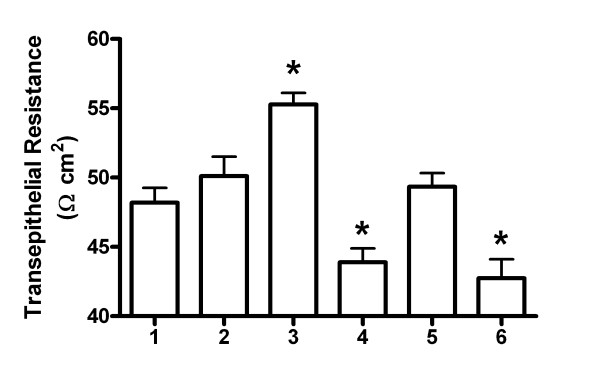
**Effect of microbes and 17β-estradiol on TEER of VEC monolayers.** Experimental groups were: 1 = VK2 cells, 2 = VK2cells + *C. albicans*, 3 = VK2 cells + 17β-estradiol, 4 = VK2 cells + 17β-estradiol + *C. albicans*, 5 = VK2 cells + probiotic lactobacilli, 6 = VK2 cells + 17β-estradiol + *C. albicans* + probiotic lactobacilli. The graph shows TEER values as mean ± standard error Ω cm^2^ for each of 6 VK2 cell monolayers. *Significantly different than group 1 by ANOVA, P < 0.05.

**Figure 2:**
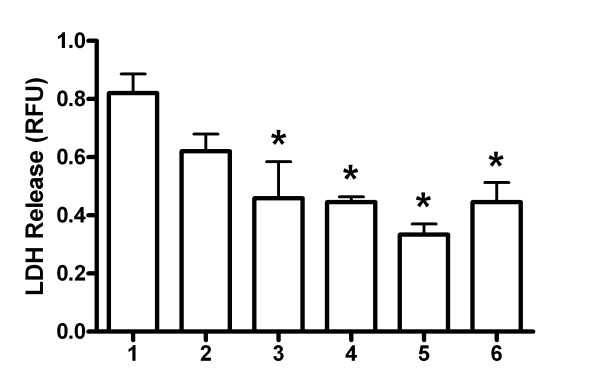
**Release of lactate dehydrogenase from VK2 cells.** The amount of LDH released into the basolateral media (shown as mean ± standard error relative fluorescence units, RFU) was compared between the following groups: 1 = VK2 cells, 2 = VK2 cells + *C. albicans*, 3 = VK2 cells + 17β-estradiol, 4 = VK2 cells + 17β-estradiol + *C. albicans*, 5 = VK2 cells + probiotic lactobacilli, 6 = VK2 cells + 17β-estradiol + *C. albicans* + probiotic lactobacilli. *Significantly different than group 1, by ANOVA, P < 0.05.

The integrity of a VEC monolayer can be disturbed by hormones and microbial activity. The presence of *C. albicans* appeared to increase the TEER of the VEC monolayers of most samples, but the effect was not statistically significant (Figure 1). When 17β-estradiol was incubated with the cells alone, there was a significant increase in TEER, but when VK2 cells were treated with 17β-estradiol and *C. albicans*-challenged, the TEER values were significantly reduced (Figure 1). The probiotic lactobacilli did not significantly alter the TEER values of VK2 cells alone or modify the reduced TEER values of VK2 cells treated with 17β-estradiol and challenged with *C. albicans* (Figure 1). These results suggest that 17β-estradiol effects on *C. albicans* infectivity may damage the cell monolayer, rather than changes in tight junctions, leading to the reduced electrical resistance. To test this, cell damage was assessed by LDH release into the basolateral medium. Infection of VK2 cell monolayers with *C. albicans* would be expected to cause some cell damage. Small amounts of LDH could be detected in the basolateral media from VK2 cell cultures. All the treatment groups (except *C. albicans*-challenged) appeared to have lower amounts of LDH in the basolateral media than VK2 cells cultured alone (Figure 2). This suggests that the treatments, including *C. albicans* contact did not induce necrosis of the VK2 cells.

### Inflammatory gene expression responses by VK2 cells to *C. albicans* are influenced by 17β-estradiol and lactobacilli

Receptors on epithelial cell surfaces detect microbes and convey the messages of detection through intracellular signal transduction pathways to nuclear transcription sites that code for response genes, such as cytokines. The expression of the genes involved in intracellular signal transduction may also be affected by cellular contact with microbes. In this study, real-time RT-PCR panels were used to measure differences in mRNA expression of microbial receptors and genes involved in intracellular and extracellular signal transduction. Table 1 shows fold-change in expression of mRNA in VK2 cells after each treatment, as compared with the level of mRNA expression in untreated VK2 cell controls. Effects on mRNA concentrations from VK2 cells treated with *C. albicans*, 17β-estradiol, and probiotic lactobacilli alone or in combinations were compared for 2-fold or greater changes in expression between columns in the table. *C. albicans* infection significantly induced VK2 cell mRNA expression of the pro-inflammatory cytokine gene IL-6 and the ELK1 gene involved in pro-inflammatory signal transduction. The presence of 17β-estradiol alone induced VK2 cell expression of IL-1β, a pro-inflammatory response. VK2 cells responded differently to 17β-estradiol when *C. albicans* was not present, as they had reduced expression of ELK1, Iκκα, IL-1α, IL-6, IL-8, TLR6, and TNFα. It caused a 2-fold decrease in expression of TLR2 by *C. albicans*-infected VK2 cells (Table 1). 17β-estradiol also did not significantly alter expression of IL-1α, IL-1β, IL-6, IL8, or TNFα mRNA expression in *C. albicans*-infected VK2 cells.

**Table 1:** Changes in expression of genes affected by experimental treatments

	**Fold changes in mRNA expression*vs.*control**				
**Gene mRNA/Function**	**Ca**	**E**_**2**_	**Ca + E**_**2**_	**Pb**	**Ca + E2 + Pb**
ELK1/Inflammatory transcription factor	+ 2.49	- 3.06	+ 1.59	- 4.46	+ 1.09
Iκκα/Induces NF-κB nuclear translocation	+ 1.68	- 1.70	+ 1.09	- 2.68	- 3.04
IL-1α/Inflammatory cytokine	- 1.64	- 2.46	- 1.39	+ 2.93	+ 3.77
IL-1β/Inflammatory cytokine	+ 1.86	+ 4.45	+ 1.64	+36.18	+ 3.80
IL-6/Inflammatory cytokine	+ 2.36	- 1.74	+ 1.16	- 1.35	- 1.01
IL-8/Inflammatory cytokine	+ 1.24	- 3.99	+ 2.59	+ 10.70	- 1.02
TLR2/Inflammatory receptor for microbes	+ 1.10	- 1.39	- 2.33	- 1.22	- 3.37
TLR6/Inflammatory receptor for microbes	- 1.09	- 5.64	- 1.93	- 2.94	- 5.08
TNFα/Inflammatory cytokine	+ 1.85	- 9.32	+ 1.37	- 5.78	- 6.78

Fold changes in mRNA expression were derived from the average values of real-time PCR threshold concentrations (n = 6, P < 0.05 by *t*-test) for each gene of interest compared to housekeeping genes. Abbreviations: (*Ca*) *C. albicans*, (E2) 17β-estradiol, (Pb) probiotic lactobacilli, an equal mixture of *L. rhamnosus* GR-1 and *L. reuteri* RC-14.

Probiotic lactobacilli induced VK2 cell mRNA expression of the inflammatory cytokines IL-1α, IL-1β, and IL-8 (Table 1). Interestingly, IL-8 mRNA induction in VK2 cells by the lactobacilli was apparently blocked by 17β-estradiol. The probiotic lactobacilli did not add to increased expression of any genes induced by *C. albicans* in the presence of 17β-estradiol. Expression of some pro-inflammatory genes was suppressed by the presence of the lactobacilli, including: ELK1, Iκκα, IL-6, TLR6, and TNFα (Table 1). The presence of probiotic lactobacilli suppressed *C. albicans* induction of ELK1, Iκκβ, and IL-6 in the presence of 17β-estradiol. Although 17β-estradiol suppressed expression of pro-inflammatory genes in VK2 cells, it did not suppress expression of pro-inflammatory genes induced by *C. albicans,* which was a feature of probiotic lactobacilli. Some cytokine genes expressed by other types of cells, such as lymphocytes, were not observed to be expressed by VK2 cells, including*:* IL-2, IL-4, IL-10, and TGFβ. The expression profile in VK2 cells resulting from the influence of probiotic lactobacilli was mostly anti-inflammatory, but lactobacilli induced IL-1α and IL-1β mRNA expression, perhaps by an alternative signal transduction response pathway to NF-κB.

### Lactobacilli suppressed inflammatory signaling protein expression

**Figure 3:**
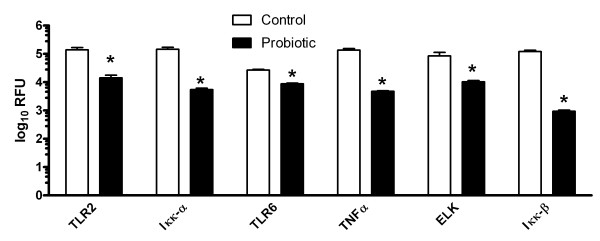
**Effects of estrogen,*C. albicans*and lactobacilli on VK2 cell expression of signal transduction and cytokine molecules.** The mean ± standard error relative fluorescence of cells from 6 experiments/group, that were stained with specific fluorescently labeled antibodies and detected by flow cytometry, are compared between (open bars) VK2 cells incubated with 17β-estradiol, challenged with *C. albicans* and (solid bars) VK2 cells cultured with 17β-estradiol, probiotic lactobacilli and challenged with *C. albicans*. *The pairs of bars were significantly different from each other (determined with paired t-tests, P < 0.05).

Effects of probiotic bacteria on expression of some of the signaling molecules and cytokines were assessed at the level of protein expression in the cells by flow cytometry. Figure 3 shows a comparison of VK2 cells expression of signal transduction proteins after incubation with *C. albicans* and 17β-estradiol with VK2 cells incubated with the probiotic lactobacilli, *C. albicans*, and 17β-estradiol. As with mRNA expression, the expression of pro-inflammatory proteins was suppressed by lactobacilli. Figure 3 shows that expression of *C. albicans*-inducible ELK1, and Iκκβ was suppressed by the probiotic lactobacilli. It also shows that VK2 cell expression of other inflammatory proteins, TLR2, TLR6, Iκκα, and TNFα that were not significantly induced by *C. albicans* were decreased by the lactobacilli.

### Probiotic lactobacilli modulated VK2 cell secretion of immune cell recruitment cytokines

**Figure 4:**
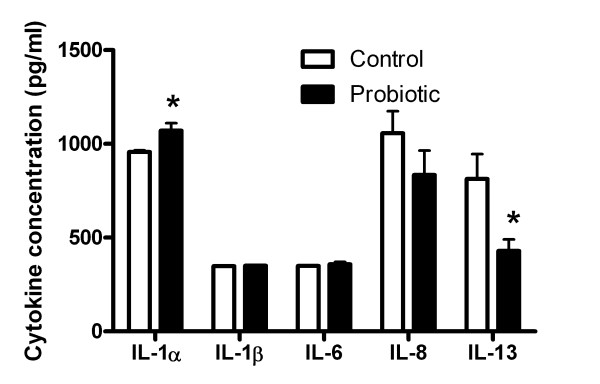
**Probiotic effects on VK2 cells secretion of cytokines.** The mean ± standard error concentrations of cytokines secreted into the basolateral medium by VK2 cells incubated with 17β-estradiol, infected with *C. albicans* (open bars) and by VK2 cells incubated with 17β-estradiol, infected with *C. albicans* after treatment with probiotic lactobacilli (solid bars) are compared. *The probiotic-treated group is significantly different from the control group by the paired *t*-test, P < 0.05.

**Figure 5:**
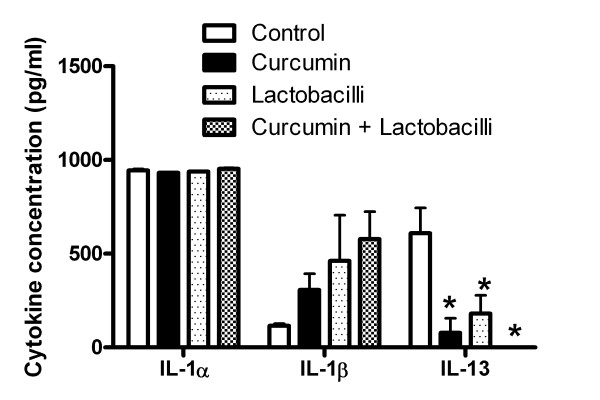
**Dependence of cytokine expression on NF-κB signaling.** The mean ± standard error concentrations of cytokines secreted into the basolateral medium by *C. albicans*-challenged VK2 cells incubated with 17β-estradiol (control), treated with or without curcumin, and colonized with or without the probiotic lactobacilli are compared. *Treatments that were significantly different from the control group by ANOVA, P < 0.05.

Since mRNA expression levels of inflammatory cytokines were affected by 17β-estradiol and probiotic lactobacilli, concentrations of those cytokines secreted into the basolateral growth medium by VK2 cells were measured with a quantitative multiplex immunoassay. The VK2 cells did not secrete detectable levels of IFN-γ, RANTES, IL-2, IL-4, IL-5, IL-10, or IL-17. The presence of 10 nM 17β-estradiol caused *C. albicans*-infected VK2 cells to secrete significantly more IL-8 from 487 ± 33 pg/ml to 1057 ± 117 pg/ml. This 17β-estradiol-induced expression of IL-8 in *C. albicans*-infected VK2 cells appeared to be blocked by probiotic lactobacilli (Figure 4). Probiotic lactobacilli significantly increased IL-1α secretion, but not IL-1β secretion by VK2 cells in the presence of 17β-estradiol and *C. albicans* (Figure 4). This effect was not inhibited by curcumin, suggesting that NF-κB signaling was not necessary to mediate these changes in expression of IL-1α (Figure 5). The probiotic lactobacilli alone significantly decreased *C. albicans*-induced secretion of IL-13 by VK2 cells (Figure 4), which was also affected by curcumin; and thus, under NF-κB-mediated regulation (Figure 5). There were no significant effects of lactobacilli on VK2 cell secretion of IL-6 (Figure 4).

## Discussion

Vaginal epithelial cells have the capacity to respond to an encounter with *C. albicans* by production of pro-inflammatory cytokines that can recruit host defenses to the infected tissue [9]. The influx of inflammatory cells results in local tissue damage that contributes to the morbidity of vaginal yeast infections. A review of clinical trials with lactobacilli to treat VVC currently reveals a lack of efficacy in curing *C. albicans* infections [16]. Wira, *et al.*[10], suggest that specific strains, like *L. rhamnosus* GR-1 and *L. reuteri* RC-14, may show some promise for treatment, but larger clinical studies are needed to know for sure.

Even if they do not cure VVC, the probiotic lactobacilli, *L. rhamnosus* GR-1 and *L. reuteri* RC-14, reduce the symptoms of vaginal yeast infections in conjunction with fluconazole treatment [17]. This suggests that there could be a host response effect by these strains beyond their role in colonization resistance. The present study was conducted to determine if there is a mechanism by which pro-inflammatory VK2 cell responses to *C. albicans* are affected by these *Lactobacillus* spp. strains. Since estrogen plays a critical role in the pathogenesis of VVC, the role of estrogen in the VK2 cell response to *C. albicans* was also evaluated.

Vaginal epithelial cells may detect *C. albicans* surface molecules with Toll-like receptors TLR2 and TLR4 [8], and possibly TLR6 [9,18], which signal through the NF-κB signal transduction pathway to initiate transcription of mRNA for pro-inflammatory cytokines. In the present study, probiotic bacteria suppressed expression of genes associated with the NF-κB signal transduction pathway and expression of TNFα and IL-8 mRNA in *C. albicans*-infected VK2 cells. Expression of TLR2 and TLR6 were induced in VK2 cells by contact with *C. albicans*, which has also been observed in normal human gingival epithelial cells infected with *Candida famata*[19]. These receptors appear to be important for epithelial cell recognition of *C. albicans*. 17β-estradiol decreased TLR2 and TLR6 expression by VK2 cells, suggesting that the hormone might leave cells less able to respond to the presence of *C. albicans* through production of immune recruitment cytokines. The presence of the probiotic lactobacilli also decreased TLR2 and TLR6 mRNA.

Since Toll-like receptors activate proinflammatory cytokine production *via* the NF-κB signal transduction pathway [8,9], it seemed pertinent to observe how expression of genes involved in this pathway and the cytokines they induce were affected by *C. albicans*, estrogen, and the probiotic lactobacilli. The increased VK2 cell expression of NF-κB-associated genes and cytokines ELK1, Iκκβ, IL-6, and TNFα by *C. albicans* challenge is consistent with induction of the TLR2/6-mediated transduction of signals through the NF-κB pathway to produce inflammatory signals, *e.g.*, IL6 and TNFα.

Vaginal epithelial cells appear to have the capacity to produce a number of different cytokines that can recruit innate and adaptive immune responses. For example, antigens from *C. albicans* induce vaginal epithelial cell line A431 production of inflammatory cytokines: IL-1α, IL-1β, IL-6, IL-8, IL-10, GM-CSF, IFNγ, and TNFα [12]. In our present study, the VK2 (E6/E7) cell line produced measurable amounts of IL-1α, IL-1β, IL-6, IL-8, and TNFα, but not IFNγ and IL-10 when challenged with *C. albicans*. IL-13 was also detected by immunoassay. Therefore, this cell line is capable of producing cytokines that can recruit innate immunity but not IFNγ and IL-10, which are mostly produced by lymphocytes. Experiments conducted with the same VK2 cell line and *Lactobacillus* spp. strains as the present study reported stimulation of IL-1α expression by *C. albicans* challenge, but not after subsequent challenge with the lactobacilli [20]. The latter study reported an increase in IL-8 expression by VK2 cells when the lactobacilli were present, which is what our present study shows in the absence of 17β-estradiol. VK2 cells responded differently to 17β-estradiol when *C. albicans* was not present, as they had increased expression of IL-1β and reduced expression of IL-8, suggesting a complicated regulatory process during the simultaneous influence of *C. albicans* and estrogen. Experimental examples show the complexity of *C. albicans* surface molecules and estrogen effects on cytokine production. It is known that bacterial surface molecules induce IL-1β, IL-6, and IL-8 in vaginal secretions [21]. *C. albicans* surface molecules induce Il-8 and TNFα mRNA by vaginal epithelial cells, which store translated cytokine proteins for secretion in a temporally dissociated manner [8]. This may explain why our experiments did not show increased amounts of IL-1β and IL-8 in the supernatants from VK2 cells with mRNA induction. Estrogen differentially regulates expression of 3000 genes in the vaginal epithelium, including the repression of IL-8 [22]. In polarized uterine epithelial cells, estradiol represses poly (I:C)-induced expression of IL-8 and reverses IL-1β induction of TNFα and IL-8 expression [23]. Similar experiments have not been described for vaginal epithelial cells until the present study.

The probiotic lactobacilli suppressed expression of NF-κB pathway-associated genes Iκκα, and ELK1, which were induced by *C. albicans* infection of the VK2 cells. They also inhibited the additive effect of 17β-estradiol on *C. albicans*-induced TNFα. These results support the role of *C. albicans* acting through Toll-like receptors and the NF-κB signal transduction pathway in VK2 cells to activate expression of inflammatory cytokines, which is suppressed by the presence of probiotic lactobacilli. Although the probiotic lactobacilli appear to suppress proinflammatory responses, their presence is associated with increased VK2 cell expression of IL-1α and IL-1β, which can recruit cellular mediators of immunity. The pro-inflammatory cytokines IL-1α and IL-1β were expressed in greater amounts as a result of the probiotic bacteria and their expression was not abrogated by the NF-κB pathway inhibitor, curcumin. An alternative signal transduction response pathway to NF-κB, such as the MAPK pathway *via* the AP-1 transcription factor, could elicit an immunological cell recruitment response to *C. albicans*. It has been shown that IL-1β is involved in the stimulation of β-defensin expression by esophageal epithelial cells in *Candida* esophagitis *via* both the NF-κB and MAPK/AP-1 signal transduction pathways [24]. The conclusion based on these results is that probiotic lactobacilli suppress the TLR2/6-initiated NF-κB-mediated induction of some pro-inflammatory cytokines, while stimulating another signal transduction pathway that induces IL-1α and IL-1β expression. The importance of IL-1β in stimulating an anti-*Candida* spp. response is exemplified by the apparent implementation of multiple signal transduction pathways for induction of its expression.

## Conclusions

The *L. rhamnosus* GR-1 and *L. reuteri* RC-14 strains are used in commercial probiotic products for maintenance of vaginal health. The present study agrees with other studies, including *in vivo* studies [1], that they inhibit infectivity of *C. albicans*, the major cause of vulvovaginal candidiasis. The results of this present study show that *L. rhamnosus* GR-1 and *L. reuteri* RC-14 may directly influence the way vaginal epithelial cells respond to *C. albicans* infections and how host defenses are recruited to the vaginal mucosa. Perhaps, the major influence on the vaginal epithelial cells from the lactobacilli is to direct the recruitment of a better host response to the site of *C. albicans* infection, one that limits damage from excessive inflammation but not the cell-mediated immune response. This study shows that the effects of the probiotic lactobacilli may be helpful, in that inflammation may be reduced, but not at the expense of immune effector cell recruitment *via* IL-1 production.

## Abbreviations

CFU, Colony-forming units; GTS, Genital tract secretions; K-SFM, Keratinocyte-serum free medium; mRNA, Messenger ribonucleic acid; MRS, deMann Rogosa Sharpe medium; PBS, Phosphate-buffered saline; qRT-PCR, Quantitative real-time polymerase chain reaction; TEER, Transepithelial electrical resistance; VVC, Vulvovaginal candidiasis.

## Competing interests

The authors declare that they have no competing interests..

## Authors' contributions

RDW conceived of and designed the experiments, conducted the statistical analyses, conducted the flow cytometry and cytokine immunoassays, and wrote the manuscript. SJJ conducted the quantitative real-time PCR analyses and cell culture procedures. All authors read and approved the final manuscript.
