# Correction: Probiotic *lactobacillus* and estrogen effects on vaginal epithelial gene expression responses to *Candida albicans*

**DOI:** 10.1186/1423-0127-19-84

**Published:** 2012-09-29

**Authors:** R Doug Wagner, Shemedia J Johnson

**Affiliations:** 1 Microbiology Division, National Center for Toxicological Research, 3900 NCTR Rd., Jefferson, AR, 72079, USA

## Text

### Correction

After publication of this article [1], we noticed that the acknowledgement of our funding source was not sufficiently specific. We hereby acknowledge that this research was supported in part by the Food and Drug Administration Office of Women's Health, USA.
